# ArtifactOps and ArtifactDL: a methodology and a language for conceptualizing and operationalising different types of pipelines

**DOI:** 10.1186/s13677-025-00761-w

**Published:** 2025-08-07

**Authors:** Raúl Miñón, Josu Diaz-de-Arcaya, Ana I. Torre-Bastida, Juan López-de-Armentia, Gorka Zarate, Lander Bonilla, Asier Garcia-Perez, Jon Aguirre-Usandizaga

**Affiliations:** 1 Digital, TECNALIA, Basque Research and Technology Alliance (BRTA), Leonardo Da Vinci, 11, Vitoria-Gasteiz, 01510 Spain; 2 Facultad de Ingeniería, University of Deusto, Avenida de las Universidades, Bilbao, 48007 Spain; 3 Digital, TECNALIA, Basque Research and Technology Alliance (BRTA), Astondo bidea, Building 700, Derio, 48160 Spain

**Keywords:** MLOps, DataOps, DevOps, Pipelines, Unified methodology

## Abstract

Machine learning is already integrated in diverse domains enhancing their performance and decision support. For laboratories, this approach is normally sufficient. However, in real environments, these models can not be generally deployed isolated since they require additional steps to satisfy an objective. These steps can range from different data transformations to the inclusion of extra machine learning models which compose an analytic pipeline. Moreover, the majority of software solutions wrap a model into an API and, rarely, focus on the whole pipeline. These are unresolved topics in the well-known MLOps methodology, specifically in packaging and service phases. In addition, these concerns can also be extrapolated to other paradigms like DevOps or DataOps.

In the context of the Pliades European project, this paper approaches the conceptualization of diverse types of pipelines from different perspectives and for different contexts, instead of simplifying the deployment and serving to an API.

Thus, ArtifactOps methodology is proposed aimed at unifying XXOps paradigms which share the majority of stages. Finally, ArtifactDL pipeline definition language is proposed to describe the key aspects identified when designing different pipelines types and to support the proposed ArtifactOps methodology. Moreover, the research presents two real scenarios to better illustrate both ArtifactOps methodology and ArtifactDL pipeline definition language and it is defined an expert evaluation conducted to validate the approach.

## Introduction

Applications involving data and artificial intelligence (AI) techniques are now widespread across various domains. Consequently, managing data and deploying AI algorithms in production environments is essential for organizations. Automating these processes with DataOps [1] and MLOps [2], which apply DevOps [3] techniques to the AI lifecycle, including feature engineering and orchestration, is highly advisable to save time on repetitive tasks [4].

When companies explore AI, they often find that data preparation involves various techniques and AI models require additional steps for deployment [5]. These steps form a data or analytic pipeline [6], which includes data acquisition, processing, preparation, and machine learning model execution. To this end, three types of artifacts are needed: data to feed the pipelines, AI models, and code for data transformations and preprocessing/postprocessing [7]. We introduce a fourth artifact type: the target infrastructure for pipeline deployment where each steps of the pipeline must be deployed. In conjunction, this allows the creation of various pipeline types, from classical CI/CD DevOps to modern DataOps and MLOps approaches, in addition to the automation of the creation and configuration of virtual machines and software installations.

However, building complex pipelines involves considering several important factors. Pipelines can be used for training or inference. Training pipelines deploy code to create a machine learning model, while inference pipelines package and deploy the model to generate results [7]. In general, artifacts are packaged using a REST API [8], hence a code application, a data transformation or a model inference are executed on demand when requested by an API client (pull interaction). Nevertheless, scenarios like the cloud continuum [9] can benefit from a streaming approach, processing data in near real time (push interaction). Regarding deployment, the whole pipeline could be deployed in the same machine or the each step composing it could be executed in a different machine.

Besides these significant considerations, pipeline governance can not be obviated in production environments. This entails the consideration of aspects like security, auditory, quality, lineage or provenance [10]. Additionally, monitoring techniques should be considered to evaluate the behaviour of the artifacts (data, models, code and infrastructure). In consequence, when a certain issue arises specific actions can be triggered. Moreover, specific security mechanisms, as well as, auditory aspects must be enabled to manage authentication and authorisation and to clearly identify the usage of involved elements. Finally, data and model quality examination is of paramount importance to deliver trustworthy applications. In this way, services can offer a high degree of reliability, security, auditory and trustworthiness extending the widely used concept of quality of service to the diverse type of previously defined artifacts. Consequently, monetization strategies can be devised under these pillars [11].

In addition, the pipeline result could potentially be exposed as a service where the nuances of his composition may be viewed as a black box. In this regard, data space producers enable the possibility to expose such result while guaranteeing interoperability, security and promoting sovereignty [12].

In recent years, cloud provider applications such as AWS SageMaker [13] or Azure DevOps Pipelines [14] have emerged to partially solve some of the aforementioned challenges. However, they tend to propose vendor-locking technologies not fully compatible with other technologies such as TorchServe [15] and TensorFlow Extended [16]. This situation complicates the use of the best software solution for each circumstance if you are conditioned to make use of an specific one for a concrete task. In addition, DevOps, DataOps, MLOps and IaC paradigms are key methodologies to deal with these artifacts. Nevertheless, the use of diverse methodologies hinders the utilisation of different type artifacts in a pipeline. This is due to the fact that in general these approaches are conceptualised to manage a specific artifact isolated (DevOps for code, DataOps for data, MLOps for models and IaC for infrastructure) but it is necessary a unified methodology which integrates the concept of a pipeline able to manage different type of artifacts. For instance, MLOps utilization is quite widespread. This is evident in works such as [17], which focus solely on MLOps, despite the potential benefits of incorporating DataOps or IaC approaches. Therefore, a unified methodology that integrates these artifacts into a single pipeline could be highly beneficial, enhancing their utilization and ultimately improving the overall work. In addition, the utilisation of pipeline description languages can help to minimize this barrier. The reason is that they tend to be independent of the underlying programming languages, infrastructure and machines and, on the other hand, flexible enough to deal with the pipelines nuances.

The work presented in this research article has been elaborated in the context of the Pliades European project [18], which focuses on AI-enabled data lifecycle optimization and Data Spaces integration for increased efficiency and interoperability. Pliades aims to develop an advanced framework for secure and efficient data sharing, emphasizing the creation of Data Spaces that connect various sectors, including mobility, healthcare, circular economy, energy, and industry. This initiative leverages AI-powered smart brokers to enhance data discovery and management, while promoting sustainable and interoperable data ecosystems. It is focused on AI-Enabled Data lifecycles Optimization and Data Spaces Integration for Increased Efficiency and Interoperability. In this direction, the contributions presented in this paper are threefold:

1. The proposition of key aspects to consider when defining different types of pipelines and a set of recommendations to guide pipeline developers.
2. The conceptualization of the ArtifactOps methodology aimed at unifying DevOps, DataOps and MLOps paradigms. This methodology does not consider artifacts as isolated elements. Conversely, it integrates them as the main entities of pipelines.
3. Definition of the ArtifactDL pipeline language devoted to conceptualise the different types of pipelines while promoting the ArtifactOps methodology.

The rest of the paper is structured as follows. “Background” and “Related work” sections describe the background work and the related works respectively. ArtifactOps methodology is explained in “ArtifactOps methodology” section. ArtifactDL is presented in “Pipeline definition language: ArtifactDL” section. “Assessment across application scenarios and by expert-judgment” section presents the evaluation. Conclusions are presented in “Conclusions” section.

## Background

**Fig. 1:**
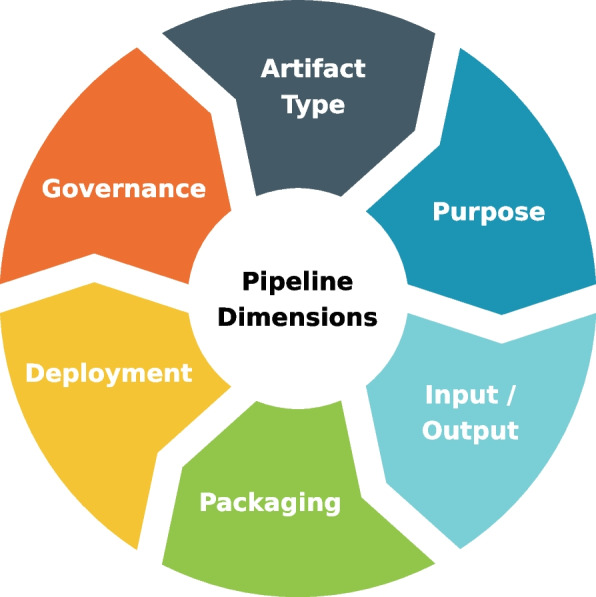
Defined pipeline dimensions

This sections presents the different pipeline dimensions (see Fig. 1) that should be taken into consideration when defining diverse types of pipelines. These aspects are the foundations of both ArtifactDL and ArtifactOps.

### Artifact type

**Fig. 2:**
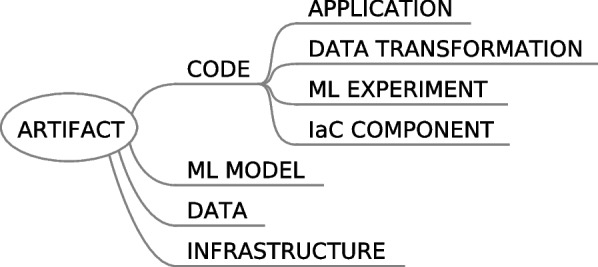
Hierarchy showing how the different artifact types are related

In “Introduction” section, four different types of artifacts have been introduced: data, code, models and infrastructure (see Fig. 2). Then, code can be materialized as an application, a data transformation, a ML experiment or an IaC component. In turn, pipelines [7, 19] are a composition of different types of these artifacts.

**Fig. 3:**
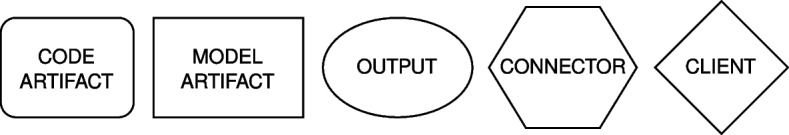
Legend for the diverse geometrical forms in the figures

**Fig. 4:**
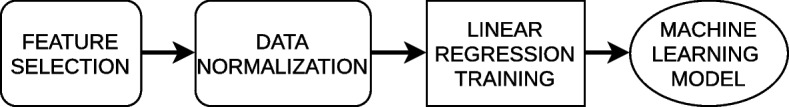
Example of a training pipeline

**Fig. 5:**
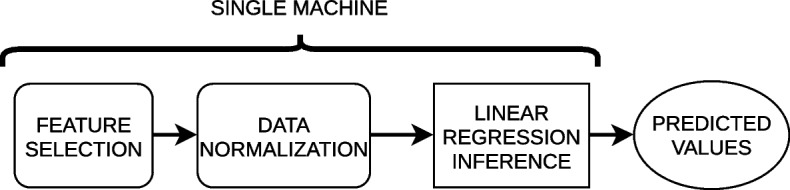
Example of an inference/analytic pipeline deployed in a single machine

**Fig. 6:**
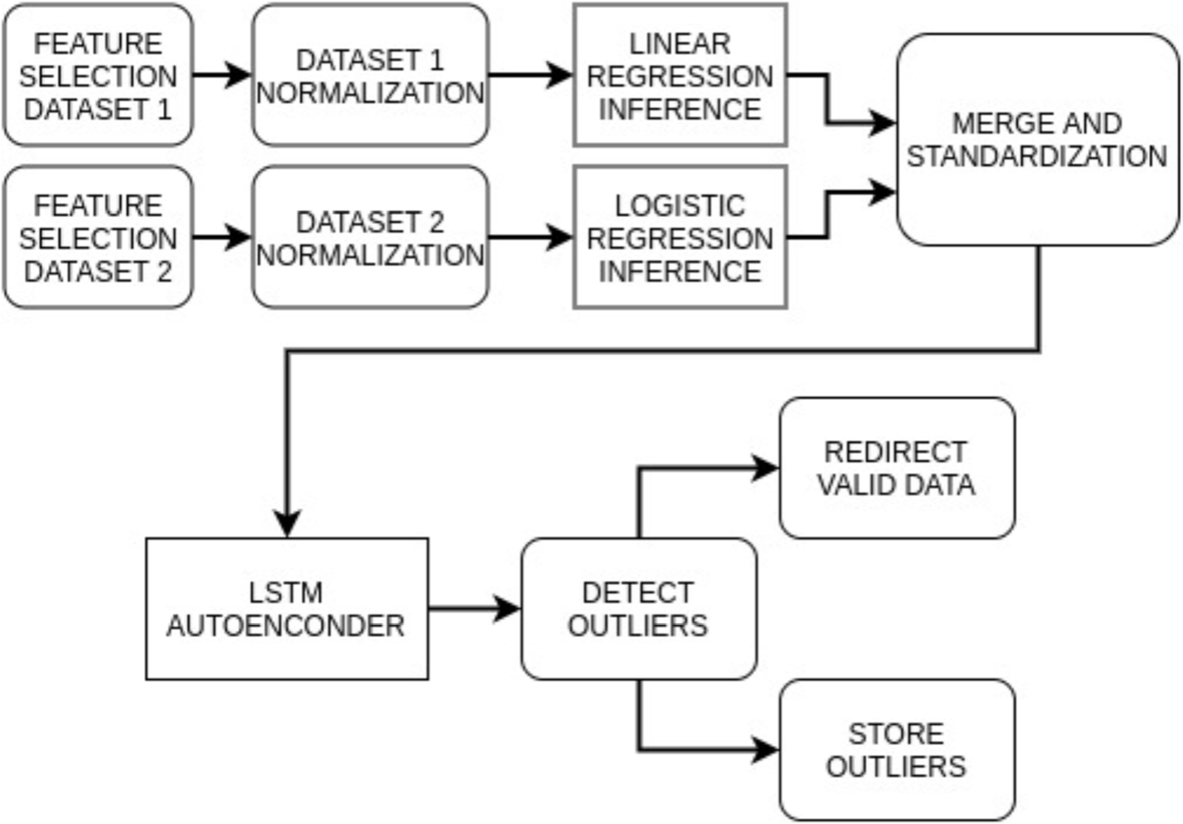
A more complex analytic pipeline following a DAG pattern

**Fig. 7:**
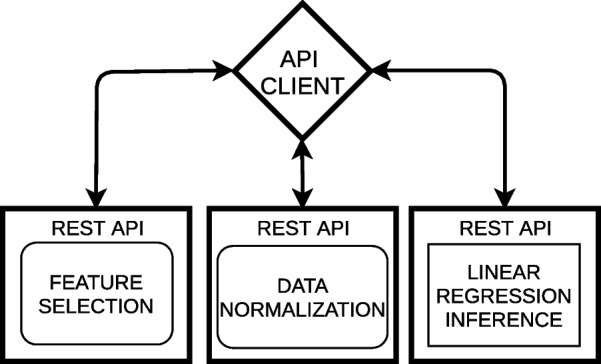
Example of a set of REST APIs wrapping each step of an analytic pipeline

**Fig. 8:**
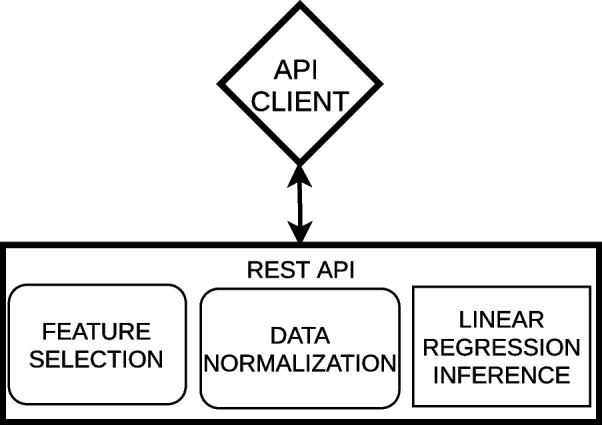
Example of an REST API wrapping the whole analytic pipeline

**Fig. 9:**
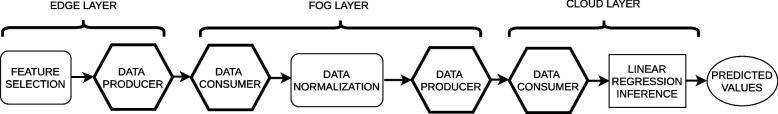
Example of a distributed analytic pipeline in edge, fog and cloud computing layers

In Figs. 4, 5, 6, 7, 8 and 9 the code artifacts are represented with rounded-corners rectangles, ML model artifacts with squared-corners rectangles, outputs of the processes with circles, connectors with hexagons and software clients with rhombus. Figure 3 summarizes this information.

### Purpose of the pipeline

The purpose or type of pipeline defines the result expected after finishing the process and drives the different artifacts that compose it. Table 1 proposes a summary of the different types of pipelines identified: CI/CD, Infrastructure as Code (IaC) [20] (a paradigm that enables programatically the automatic management of the infrastructure by using code), Data, Train and Inference/Analytic. Fig. 4 shows an example of a training pipeline, which produces a linear regression model, while Fig. 5 represents the previously generated model deployed to predict values. However, more advanced pipelines could be composed like the example presented in Fig. 6.

**Table 1:** Purpose of the different types of pipelines

Type of pipeline	Purpose	Expected result
CI/CD	Deploy applications in a production environment	One or various code artifacts representing applications deployed in a set of machines
IaC	Programatically creation and/or configuration of machines	Machines adequately created and configured
Data	Apply transformations over a set of data which can involve aspects like ingestion, preparation or quality validation	A set of code artifacts representing data transformations running to orchestrate these tasks
Train	Execute the steps required to create a ML model (data preparation, feature engineering, training, validation)	A set of code artifacts representing these ML training phases running to orchestrate these tasks (special code artifact for training)
Inference/Analytic	Put in production an analytic pipeline including models to perform inferences and the corresponding pre and post processing steps	A set of code and model artifacts running to orchestrate inference processes

There are methodologies focused on managing and automating the life-cycle of these types of pipelines. Regarding CI/CD, DevOps [3, 21] was the original and has served as an inspiration for the rest of them. It aims at reducing the gaps between development and operation teams and to offer continuous deliveries by automatizing software life-cycle. IaC permits the reduction of deploy times, costs and errors while the infrastructure consistency is guaranteed among the diverse machines [22]. For example, Vagrant [23] permits the automatic creation of virtual machines and Ansible [24] the automatic configuration and software provisioning on remote machines. Meanwhile, DataOps [1, 25] is focused on the integration and automation of data flows, by using ingestion, preparation, orchestration or data quality techniques. Finally, train and inference life-cycle is automated by the use of MLOps principles [26–29].

The study of these paradigms have allowed the better understanding of all of them to clearly identify their nuances. This has enabled the conceptualization of the ArtifactOps methodology to unify them while considering pipelines of diverse artifacts.

### Input/output elements

On the one hand, CI/CD and IaC pipelines do not require any specific input, only the code to carry out the task. Conversely, their outputs are the application deployed and the machines prepared respectively. On the other hand, Data, training and analytic pipelines are fed with data, but while data and analytic pipelines output also data, the training process result is a ML model. Additional considerations should be considered. For example, whether the data is streaming and, consequently, should be processed with a continuous pipeline or periodically or how to deal with the versions of the artifacts generated.

### Package mode

The packaging of a pipeline considering different types of artifacts and technologies can be a technical challenge. However, a robust methodology based on appropriate wrappers or data exchange formats, helps in overcoming these problems and achieving effective integration between different artifacts. The interaction could be conducted using the pull or push communication patterns.

In pull mode, each artifact (see Fig. 7) or the whole pipeline atomically as a unique service [30] (See Fig. 8) can be wrapped in a REST API, but other alternatives like serverless technologies can be utilized. In this category, Amazon’s Lambda [31], Azure Code Functions [32] and Google Cloud Functions [33] provide specific techniques to wrap code functions from diverse programming languages. ArtifactDL is based on this principle. The idea is that you can provide your artifact as a black box but it is mandatory to define key meta-information to manage it and to make it interoperable with other artifacts by providing a common syntax. In this pattern, a software client is required to trigger the pipeline process.

Conversely, in the push pattern a process listens the arrival of new data, which is processed in real time or near real time (see Figs. 4, 5 and 9). This options is key for streaming scenarios like a big data platform automating the incoming data or for continuous training pipelines being feed with incoming data.

Another possibility is to package the whole pipeline and offer it as a service to a bigger ecosystem like in a data space [34]. In this regard, efforts like GAIA-X [12] and International Data Spaces (IDS) [35] provide support for specific connectors [36] that could abstract the nuances of the packaging and deployment of the pipeline like the Eclipse Dataspace Connector [37]. Examples of functional and active data spaces are CATENA-X [38] in the automotive domain, EUROPEANA [39] at a cultural domain, or EONA-X [40] in energy data.

### Deployment mode

The whole pipeline can be atomically deployed in the same target machine (see Fig. 5) or each step in diverse machines in a distributed fashion [30]. This type of pipeline is really useful in cloud continuum architectures where the infrastructure is composed of edge, fog and cloud nodes or even on-premise nodes. In this way, not very resource-demanding steps or steps involving sensible data can be deployed in edge and fog layers and the heavier ones in the cloud or dedicated servers. It is worth mentioning that distributed pipelines requires additional mechanisms to send and receive data among the diverse machines involved. This is not the case with atomic pipelines because the output of an step can be automatically redirected to the input of the following one. For this purpose, APIs, message queues or specific technology connectors must be used like it is exemplified in Fig. 9.

Furthermore, additional derivations of atomic and distributed pipelines could be useful for specific contexts:

- When deploying the steps by using a container technology, in atomic pipelines the distinct steps could be deployed in the same container or in different ones. In the later case, despite being in the same machine, the pipeline should be considered as a distributed one since the same communication mechanisms must be provided to enable the data flow.
- Steps of a pipeline could be horizontally distributed, in parallel, in distinct machines following a Big Data paradigm when managing or a replica mechanism for applications. Thus, the workload of the step can be divided into the target machines.
- Federated Machine Learning is a specific case of training a distributed analytic pipeline since the nodes where models are deployed must coordinate with a centralized node in order to aggregate the all resultant models after their training phase.

### Governance

The concept of artifact governance involves among other aspects to consider the quality and security of code, data, infrastructure and models [10]. Regarding data quality, Strong et al. [41] defined several key dimensions to consider: accuracy, timeliness, completeness, consistency, objectivity, believability and relevance. Janssen et al. [42] propose a framework for addressing data governance for trustworthy artificial intelligence. Some of these principles are crucial for the present work and have also been considered for building ArtifactDL governance aspects such as *evaluate data quality and bias*, *detect changing patterns* or *minimize authorization to access data*.

As security is concerned, relevant open source tools like Keycloak [43] enable the management of the identity of users and services, as well as the possibility of creating authorization policies to enhance the access control. Another aspect to concern is the privacy of the data utilized which must be preserved when necessary.

In addition, auditory mechanisms can be studied to better understand deployed pipelines in production environments and enable monetization services. Cloud providers and intermediaries help companies abstract cloud usage and offer machine learning as a single service (MLaaS). To achieve this, services must be conceptualized and accessed via APIs or streaming mechanisms for auditing. Key monetization points in MLaaS are outlined in [44], which has been useful for integrating monetization aspects in operationalizing ML models and artifacts.

## Related work

There exist a number of languages for boosting model interchange formats. They focus on defining machine and deep learning models independently of target environments, programming languages or frameworks. In this way, models can become portable to a wider variety of contexts. For this purpose, an engine capable of recognizing and executing the language syntax must be provided to be integrated in applications. Additionally, sometimes they also support the inclusion of preprocessing and postprocessing steps to enable simple pipelines. In this field, PMML [45] is a model interchange format to define predictive and descriptive models and their preprocessing and postprocessing steps. It targets at easing the migration of models between environments while avoiding vendor locking issues. Portable Format for Analytics (PFA) [46] is a language focused on defining analytic models independent of the underlying tools, applications or systems. Thus, the model can be deployed in diverse target environments facilitating the integration with software products. Moreover, it also enables the definition of data transformations and aggregations to materialize pre and post processing steps. When a PFA model is produced, its scoring engine (a software library developed for both Java and Python) can be used to score results. PFA states to overcome PMML when preprocessing and postprocessing features is concerned, in addition to not having to update the scoring engine to integrate new models. Open Neural Network eXchange (ONNX) [47] is an open format for representing machine and deep learning models. It provides a set of building blocks and a format compatible with diverse frameworks, engines and tools like Scikit-learn [48], TensorFlow [49] or PyTorch [50]. These languages share certain similitude with [51] ArtifactDL because they can define analytic pipelines in some extent. However, ArtifactDL is not focused on defining the specific model mathematical representation and related operators. Conversely, it provides a full syntax for defining the steps of analytic pipelines alongside meta-information for characterizing it, but referring external previously created artifacts. Moreover, other pipelines like training or data pipelines can be defined and a set of additional contextual properties like the target infrastructure or governance aspects.

In the DevOps field, among others, there are two key types of tools interesting for this research. On the one hand, tools that permit the creation of CI/CD pipelines to iteratively test and deliver software projects into production environments by managing the software development life-cycle. Belonging to this family, successful tools are Jenkins [52], CircleCI [53], GitLab CI/CD [54] or cloud services like Azure Pipelines [55] and AWS code pipelines [56]. On the other hand, software orchestrators, based on container technologies and/or IaC tools, which programatically manages the application workflows, deployments or the infrastructure. In this area, on the one hand, Verma et al. [57] propose a cluster manager to operationalize workflows at big scale. This work served as Kubernetes’ foundation [58], which is a widely adopted solution on the industry. Another possibility is the utilization of Docker Swarm [59], which has less features but, in turn, the computational cost is reduced. On the other hand, Ansible [24] enables infrastructure automation through an agentless approach, simplifying configuration management. Vagrant [23] provides an efficient way to manage virtualized environments, ensuring consistency across development and production setups. Terraform [60] allows infrastructure provisioning using declarative configurations, making it easier to manage infrastructure as code at scale. Pangea [6] automatically generate and provision execution environments for analytic pipelines across edge, fog, and cloud layers, thereby simplifying the deployment process for users. These DevOps technologies have been proved to be tremendously helpful for IT professionals. Nevertheless, their objective relies only on deploying code artifacts into a target machine. Excepting Pangea, but is only focused on analytic pipelines. The life-cycle management of models and data is not considered.

Regarding data life-cycle management, DVC pipelines [61] is a command line tool that allows to chain different code projects for creating data pipelines to perform ETL operations. To this end, some properties can be adjusted such as the output, the entrypoint or a GIT URL as the project source. Additionally, ML training pipelines are also supported. Apache Airflow [62] and Prefect [63] are software tools that programatically allow create, schedule and monitor batch ETL pipelines or workflows by using Python. Nonetheless, like DevOps tools, they are specially focused on only managing one type of artifact. Data in this case, obviating other pipeline types like analytic pipelines.

The same situation is repeated when analyzing MLOps tools. They are only focused on model artifacts. In this field, MLFlow Pipelines [64] enable the possibility to create training pipelines, package and deploy the resulting model into a production environment. This solution covers a wide range of stages of the ML life-cycle. However, similarly to CI/CD pipelines it deploys a resulting artifact into a single machine. Conversely, ArtifactDL manages the whole pipeline to deploy, considering the packaging and deployment of other artifacts needed to compose the solution like preprocessing and postprocessing steps. Additionally, it put the focus on the complete ML life-cycle including other missing stages in MLFlow like the monitoring and the re-deployment. Seldon [65] is an advanced MLOps solution that offers features for serving, explaining and monitoring, as well as, including some governance capabilities but neither the management of diverse artifacts as a whole nor the deployment of them in a distributed fashion are taken into account.

Summarizing, the conceptualization of ArtifactOps methodology and ArtifactDL pipeline definition language deserved to be conducted. In Table 2, we can see that most of the analyzed works and tools lack features for managing different types of artifacts. Additionally, only a few of them integrate the concept of a pipeline as a flow composed of multiple artifacts. In addition, also few of them considers governance aspects and specific packaging (like streaming) and deployment (like distributed) modes. Therefore, there exists a gap for the creation of ArtifactDL to put these functionalities together while avoiding the use of multiple dispersed tools, in addition to the creation of ArtifactOps methodology to guidance developers adopting this approach. The following section compares the different DSL tools available, highlighting their strengths and features in addressing the needs identified in this research.

**Table 2:** Comparison of tools that boost the deployment of different type of pipelines

Framework	Analytic pipelines	CI/CD pipelines	IaC pipelines	Data pipelines	MLOps pipelines
PMML [45]	$\checkmark$	✗	✗	✗	✗
PFA [46]	$\checkmark$	✗	✗	$\checkmark$	✗
ONNX [47]	$\checkmark$	✗	✗	✗	$\checkmark$
Jenkins [52]	✗	$\checkmark$	✗	✗	✗
CircleCI [53]	✗	$\checkmark$	✗	✗	✗
GitLab CI/CD [54]	✗	$\checkmark$	✗	✗	✗
Azure Pipelines [55]	✗	$\checkmark$	✗	✗	✗
AWS Code Pipelines [56]	✗	$\checkmark$	✗	✗	✗
Kubernetes [58]	✗	✗	$\checkmark$	✗	✗
Pangea [6]	$\checkmark$	✗	$\checkmark$	✗	✗
Terraform [60]	✗	✗	$\checkmark$	✗	✗
Ansible [24]	✗	✗	$\checkmark$	✗	✗
Vagrant [23]	✗	✗	$\checkmark$	✗	✗
Docker Swarm [59]	✗	✗	$\checkmark$	✗	✗
DVC [61]	✗	✗	✗	$\checkmark$	✗
Apache Airflow [62]	✗	✗	✗	$\checkmark$	✗
Prefect [63]	✗	✗	✗	$\checkmark$	✗
MLFlow Pipelines [64]	$\checkmark$	✗	✗	✗	$\checkmark$
Seldon [65]	$\checkmark$	✗	✗	✗	$\checkmark$
ArtifactDL	$\checkmark$	$\checkmark$	$\checkmark$	$\checkmark$	$\checkmark$

$\checkmark$: full support, ✗: not supported

### DSL tools to improve pipelines

Several DSL tools have been developed to improve CI/CD pipelines, data science workflows, and domain-specific applications. StalkCD enhances interoperability between CI/CD pipeline formats, providing support for Jenkins and BPMN with minimal information loss and facilitating workflow visualization [66]. SPaaS offers an extensible DSL-based pipeline generator for Jenkins, allowing custom activities to be incorporated while enforcing governance policies to standardize development practices across teams [67].

A model-driven framework for data science workflows improves reproducibility by separating conceptual and operational concerns, enabling the use of diverse tools, and integrating model verification rules to maintain workflow consistency [68]. Forma DSL optimizes image processing pipelines for CPUs and GPUs, leveraging compile-time analysis for efficient hardware utilization and seamless integration with Python and OpenCV [69]. NLDSL simplifies DSL implementation by bundling syntax descriptions and operation execution within Python functions, offering enhanced usability with IDE support, code completion, and the ability to embed DSL statements directly in source code comments [70].

The SciVi platform applies ontology-driven methodologies to automate the development of visual analytics software, ensuring validity while maintaining an extensible repository of domain-specific ontologies [71]. Ziria DSL is designed for wireless physical layer tasks, implementing specialized optimizations such as lookup table generation and pipeline fusion to enhance performance, achieving execution speeds comparable to or surpassing hand-tuned C++ implementations [72]. Table 3 clearly highlights ArtifactDL from the rest of the approaches due to its versatility to deal with several pipelines types. Conversely, the other works identified only support one type of pipeline.

**Table 3:** Comparison of domain-specific language tools for pipeline

DSL tool	Use case	Advantages	Key features
StalkCD Framework [66]	CI/CD pipelines	High interoperability, empirical support for Jenkins files	Interoperability, Jenkins and BPMN support
SPaaS [67]	Jenkins pipelines	Customizable, supports organizational policies	Extensible, template-based, governance enforcement
Forma DSL [69]	Image processing pipelines	High efficiency, easy integration with popular tools	Targets CPUs and GPUs, efficient code generation, Python/OpenCV integration
NLDSL [70]	Data science pipelines	Simplifies DSL creation and maintenance, supports multiple IDEs	Simplified implementation, IDE support, flexible embedding
SciVi Platform [71]	Visual analytics pipelines	Guarantees validity, supports various application domains	Ontology-driven, automated development, extensible repository
Zirira DSL [72]	Pipeline reconfiguration	High performance, flexibility in wireless protocol implementation	Specialized optimizations, high performance, pipeline reconfiguration
Model-Driven Framework [68]	Data science pipelines	Enhances reproducibility and replicability, ensures consistency	Conceptual and operational layers, reproducibility, verification rules
ArtifactDL	CI/CD, IaC, Data, train and inference pipelines	Simplifies complex pipeline design by abstracting technical details into a unified language while consider a wide range of aspects	Modular, declarative, and extensible language with built-in support for traceability, governance policies, and artifact portability.

## ArtifactOps methodology

**Fig. 10:**
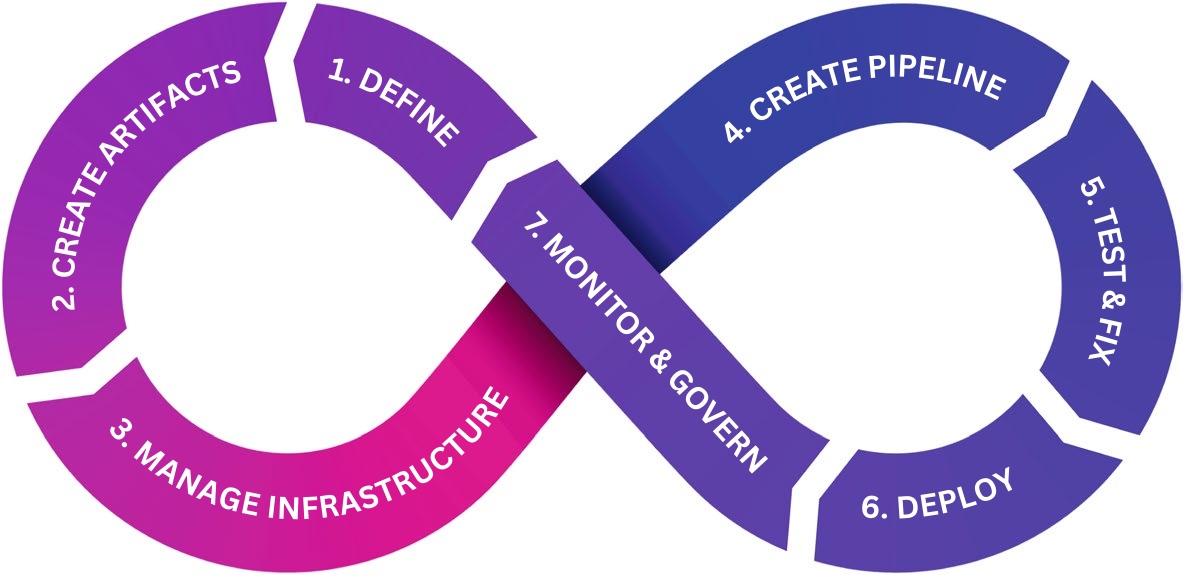
ArtifactOps methodology life-cycle

This section presents ArtifactOps methodology, which is illustrated in Fig. 10. This methodology has been defined by analysing the diverse XXOps approaches (DevOps, MLOps and DataOps), identifying their main differences, generalizing some related concepts and unifying all of them in a unique methodology. It aims at providing a set of useful steps to follow when conceptualising a code, data or analytic pipeline. It is worth noting that depending the studied source the stages can slightly vary. However, after reviewing several scientific papers and related information (like those on the background and state of the art), we have tried to set the most used stages based on recent scientific literature to mitigate this issue. Concretely, the steps selected are based on the following sources: DevOps [21], MLOps [28] and DataOps [25]. It should be also noted that XXOps methodologies normally only deal with a specific artifact like a software in DevOps or a ML model in MLOps. Conversely, this methodology takes the whole pipeline into account.

Unlike traditional XXOps methodologies, where infrastructure configuration is often treated as an implicit or supporting task (e.g., embedded in CI/CD pipelines or IaC scripts), ArtifactOps explicitly includes *Infrastructure Management* as a distinct and standalone phase. This decision is based on the increasing complexity and modularity of modern pipelines, which require formal treatment of infrastructure as a versionable, testable, and governable artifact. To this end, IaC [20] paradigm is embraced.

In addition, it can be noticed that DevSecOps [73] approach is also considered in the governance phase. AIOps [74] is not explicitly mentioned because it relates to the application of AI techniques to IT Operations. Consequently, in this case it will depend on the content of the artifacts utilised in a pipeline.

**Table 4:** Matching phases of different XXOps methodologies with ArtifactOps

ArtifactOps	DevOps	DataOps	MLOps
Definition	Plan	Plan	Model Requirement
Artifacts Creation	Develop	Create	Data Collection Data Preparation Create Feature Engineering Model Training
Infrastructure Management			
Pipeline Creation		Orchestrate	
Test and Fix	Verify Test	Test and Fix	Model Evaluation
Deploy	Deploy	Deploy	Deploy
Monitor and Govern	Operate Monitor	Operate Monitor Optimize Feedback	Monitor

Table 4 presents the stages of each methodology providing a single vision to clearly check the similarities and differences. Moreover, the first column lists the proposed stages for ArtifactOps as a unified approach, which are defined below:

1. *Definition:* This phase states the pipeline objective, clearly establish a set of requirements and defines the sub-phases to consider:
  - Ecosystem and context analysis: Depending on the context in which the pipeline needs to be integrated and deployed, different approaches may be recommended. Therefore, it is highly advisable to consider this from the very beginning of the conceptualization process.
  - Pipeline definition and characterization: this implies the identification of the diverse artifacts involved in the pipeline, the input and output of all of them, the type of artifact (code, training algorithm or model) and the required artifact and pipeline tests.
  - Infrastructure analysis: a study of both the available and required infrastructure has to be carried out. This serves to determine whether the company resources are enough or additional ones must be provisioned and to better understand the pipelines requirements.
  - Governance aspects must be carefully studied to deploy pipelines in production environments. As security is concerned, roles, users and its permissions have to be defined, as well as the techniques to provide such mechanisms. The tracking of artifacts and input and output sources must be also defined to guarantee a successful auditory system. Moreover, the determination of quality metrics is necessary to be able to analyse the good behaviour of the elements involved: models, code, data and infrastructure.
  - Having established the governance aspects, it is recommended to define which artifacts will be monitored and in which extent.
  - With the whole picture defined, final decisions must be conducted like the package and deploy modes and the specific flow of the pipeline.
  - *Expected output:* pipeline definition documentation
2. *Artifacts creation:* in the distinct approaches analyzed appear tasks like develop, code, build, model train and feature engineering. In essence, all of them belongs to the category of artifact creation. Therefore, we have generalized this stage to this concept to integrate all these phases. The main idea consist in developing the necessary traditional code, data transformations (also as code), training algorithms, final ML models, artifact tests and acquire the necessary data. Besides functional pipeline steps, required artifacts for dealing with governance aspects can not be obviated. Finally, the artifacts should be managed by a version control management system. It is recommended, for instance, to utilize Git with DVC to track the changes of the created artifacts, regardless their size, and being able to deal with the different versions, enabling agile modifications and team collaboration. It should be noted that steps like data collection or data preparation are directly integrated in this stage since we consider them as pieces of code (that is to say an artifact) which performs a specific data operation/transformation.
  - *Expected output:* all the artifacts developed and the data required acquired.
3. *Infrastructure management:* before the deployment processes the creation and adequate configuration of the target infrastructure must be accomplished for both testing and production environments. To this end, it is highly advisable to follow the IaC pattern creating an additional pipeline of chained IaC component artifacts.
  - *Expected output:* infrastructure created for both test and production environments.
4. *Pipeline creation:* this step entails putting all the artifacts together to conform the final pipeline and by using the previously created artifacts and the infrastructure. To this end, it is recommended to use a syntax (independent of the programming languages, technologies, frameworks and target environment) with enough expressiveness to characterize adequately the artifacts, their governance aspects, the pipeline flow, monitoring and package and deploying modes. A syntax with these objectives is presented in “Pipeline definition language: ArtifactDL” section. This defines orchestration of the pipeline by setting unequivocally the dependencies between artifacts and the target deployment infrastructure. Finally, the required pipeline tests must be also defined.
  - *Expected output:* pipeline totally defined including the target infrastructure.
5. *Test and Fix:* this phase involves the deployment in the testing environment of both the artifacts isolated and the whole pipeline. Then, the artifact tests to verify the required functionalities and the pipeline tests to check the good behaviour of the flow and the compatibility among artifacts are executed. Finally, the issues identified must be fixed and this stage must be executed again until being successful. Depending on the artifact different strategies can be followed to perform the required tests. For instance, code can be tested by the use of unit testing frameworks like PyTest [75], JUnit [76], etc, data using tools like Great Expectations [77] or DQDF [78] and ML Models with model evaluation metrics, bias tests or performance benchmarking using libraries like Scikit-learn [48], MLflow [64], or TensorBoard [79], etc.
  - *Expected output:* artifacts and pipeline behaviour validated.
6. *Deploy:* Having adequately tested the artifacts and the pipeline, in this phase the whole pipeline is deployed in the specific mode defined in the production infrastructure. This process will be in charge of parsing the pipeline definition document and conducting the required tasks like download each artifact from the repository to the production environment, package them as specified in the pipeline definition and serve them to be usable and ensuring the governance established.
  - *Expected output:* pipeline deployed in the production infrastructure.
7. *Monitor and Govern:* these two tasks are set together because of their tight intersection. Artifacts must be adequately monitored to guarantee that the pipeline objective and their requirements are guaranteed not only when the pipeline was deployed but also in the future. The same happens with input and output elements. For instance, there could be a deviation in the input data because of a sensor failure or the resulting model of training pipeline is not behaving as expected. Monitoring the infrastructure is also crucial to maintain the level of service. Finally, governance aspects like security must be also monitored but aspects like auditory depends intrinsically of the monitoring system. On the other hand, specific actions must be programmed when certain state happen during the monitoring phase like triggering an alarm, redeploying a pipeline or deploy another pipeline. For instance, when a training pipeline finishes an inference pipeline could be deployed.
  - *Expected output:* a working mechanism to guarantee the good behaviour of all the involved elements including both functional requirements and non functional like governance aspects.

These phases will be iteratively executed triggering in the subsequent iterations only the required ones. For example, an artifact could be modified due to a bug detected while monitoring. This does not imply to modify its definition but yes to test and deploy it again.

The adoption of ArtifactOps involves not only technical alignment but also significant organizational adaptation. As the methodology unifies practices from DevOps, MLOps, DataOps and IaC, it requires breaking down traditional silos and fostering collaboration across software engineering, data engineering, and operations teams. To facilitate this, organizations are encouraged to form interdisciplinary teams with effective communication and supported through regular meetings. Additionally, successful implementation depends on proper training programs that cover the unified life-cycle principles, governance mechanisms and derived tools.

### Tooling ecosystem for ArtifactOps

Although ArtifactOps is a conceptual and technological methodology agnostic to specific tools, its effectiveness and adoption heavily depend on the availability and integration of adequate tooling. The methodology is built around concepts like defining, versioning, deploying or governing artifacts in a structured way, which is operationalized via ArtifactDL-an expressive, declarative language that describes elements like pipelines, artifact metadata, governance requirements or infrastructure. Consequently, to support ArtifactOps, the following components are required:

- *ArtifactDL-compatible tools*:
  - *Editor*: A YAML/JSON/DSL editor with syntax highlighting and schema-based autocompletion (e.g., VSCode with custom extensions).
  - *Validator*: A CLI or web-based validator that ensures ArtifactDL definitions conform to a schema.
  - *Execution Engine*: A runtime that parses the ArtifactDL definition and orchestrates the deployment using tools like Docker or Kubernetes.
  - *Monitoring Agent*: A monitoring component that reads the observability configuration in ArtifactDL and connects to Prometheus, Grafana, or OpenTelemetry-based systems.
  - *Integration with other existing tools* like GitLab [54], DVC [61], MLFlow [64], Great Expectations [77], DQDF [78], Terraform [60], Ansible [24], Airflow [62], PyTest [75], JUnit [76], CI/CD tools, etc.

While parts of this ecosystem can leverage mature open-source tools, others, particularly those tightly coupled with ArtifactDL are under development.

### Applicability across different domains

The ArtifactOps methodology is designed to be modular and adaptable, making it applicable across a broad range of domains. Particularly it becomes specially significant in safety-critical environments (e.g., healthcare, aerospace, autonomous systems), where compliance with rigorous standards and regulations is mandatory. Concretely, the operationalization of ArtifactOps phases like “Governance” and “Test and Fix” can be extended to include formal verification, regulatory checklists, traceability matrices, and safety cases, allowing alignment with existing certification processes.

Conversely, in less regulated environments (e.g., startups, experimental labs), the methodology can be applied in a more lightweight fashion, using simplified governance models and prioritizing agility over formality. In such cases, the governance sub-phase may include minimal role definitions, lightweight versioning, and informal testing protocols. This flexibility ensures that ArtifactOps does not impose unnecessary overhead while maintaining structured lifecycle stages.

## Pipeline definition language: ArtifactDL

In 2020, PADL (Pipeline definition And Deployment Language) was presented [51] aimed at defining the different steps required to deploy an analytic pipeline considering target machines. For this purpose, an elaborated schema was provided to define key aspects of the analytic pipelines such models involved in the pipeline, input and output queues for each step of the pipeline or underlying constraints for the deployment. Figure 20 in the Appendix shows the original schema defined and presented in [51].

**Fig. 11:**
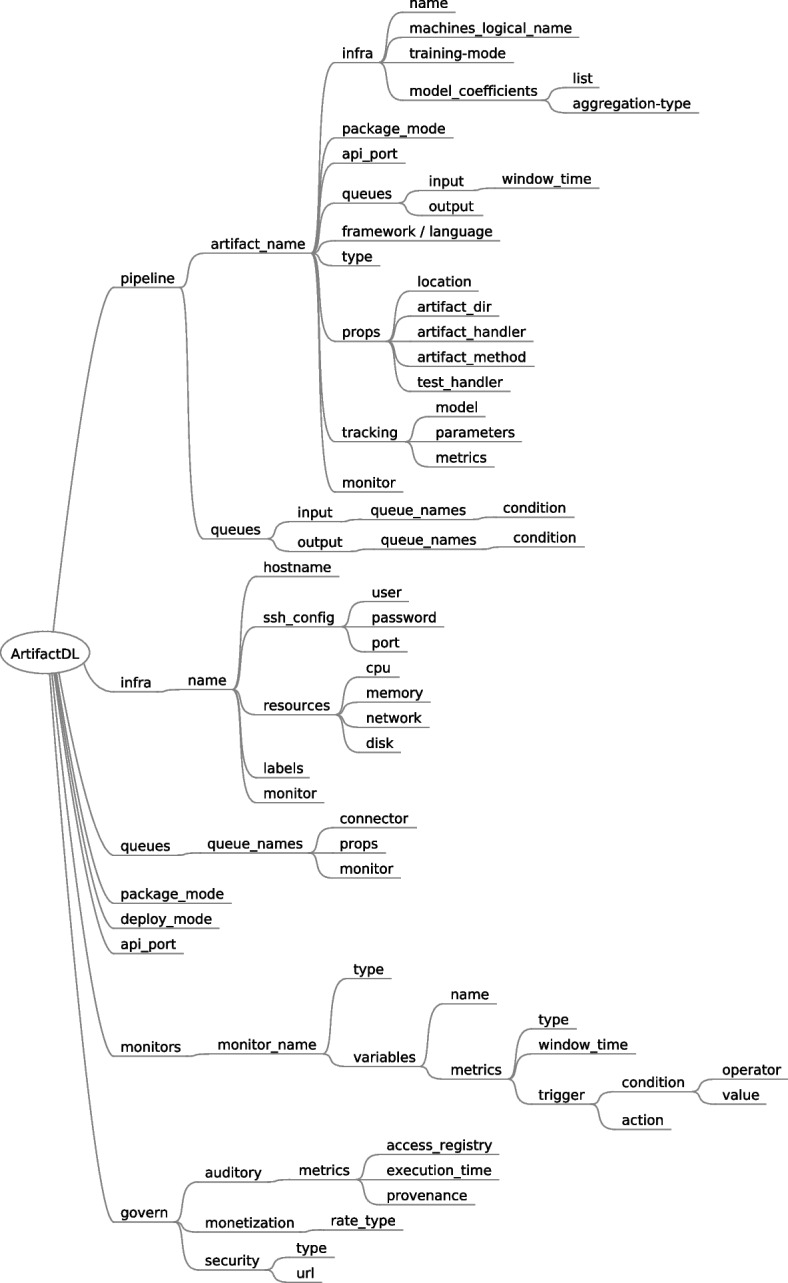
Tree Diagram showing the ArtifactDL Schema

Now, three years later and after being used in a number of projects such as [6, 30, 80], we have created a new language denominated ArtifactDL. Instead of only managing analytic pipelines until a reduced extent, it is able to manage diverse types of pipelines and significant different aspects ArtifactDL schema is showcased in Fig. 11. Below the main new features are listed, alongside scientific articles justifying its conceptualization, and the following subsections provide additional details:

- Flexible management of the infrastructure and enable direct relationships between machines of the infrastructure and pipeline steps [81].
- Possibility to define various input and output queues and output selection based on a conditional rules [82].
- Support for different types of pipelines integrating the possibility to select the *package* and *deploy* modes [83].
- Deployment of not only models but also code applications which enables support for a wider variety of pipelines [84].
- Detailed support for training pipelines [85].
- Support for monitoring infrastructure, data and models [86–88].
- Data governance features like security, auditory, data and model quality and monetization [89, 90].

### Infrastructure support

**Fig. 12:**
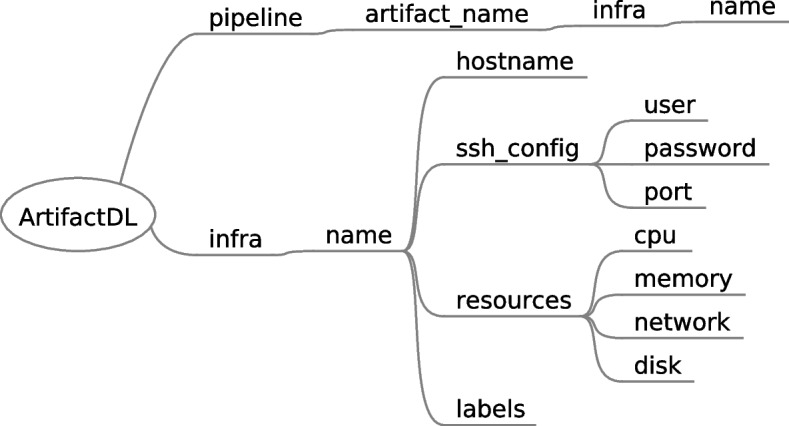
Syntax included in ArtifactDL for defining infrastructure concepts

PADL allowed to specify infrastructure constraints when defining the deployment of a step of an analytic pipeline. Now, ArtifactDL includes support for targeting specific machines to deploy the pipelines. Figure 12 shows the new designed elements. On the one hand, *infra* element under root element (*ArtifactDL*) defines the diverse machines available. For this purpose, initially a logical name is assigned to enable its reference from pipeline steps. Next, the following properties are supported:

- *hostname* of the machine
- *ssh_config* for defining a mechanism to programatically manage the machines allowing the deployment of the artifacts.
- *resources* for characterizing the machines
- *labels* to define additional relevant information. For instance, it could be stated that a machine belongs to the edge layer or to the testing or production environment.

On the other hand, each artifact can reference a specific machine (or a set of machines like it is explained in “Training pipelines” section) by using the logical name defined under its own *infra* element. This way, it is quite straightforward to set the machines for the testing or for production environment. For this purpose, the use of a GIT branch is recommended to easily manage the modification of a pipeline description from testing to production.

### Flexible queues

Analytic pipelines must not necessarily provide an straightforward path. As exposed in Fig. 6, they can be structured, for example, as a direct acyclic graph with multiple paths. This requires a certain degree of flexibility when defining input and output queues. For this purpose, ArtifactDL supports multiple inputs and outputs to be able to merge different paths in the same element or to redirect the output to multiple elements. In addition, conditional output mechanism offers the possibility to send data to one entity or other considering a specific output of the process.

**Fig. 13:**
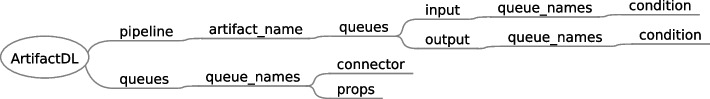
Syntax included in ArtifactDL for defining queue concepts

Figure 13 shows how the *queues* element (extending from *ArtifactDL* root element) defines the connector required for a specific queue and the required properties to use it for distributed mode. Then, each artifact element (under its own *queues* element) can define a set of inputs and outputs. In distributed mode, a queue element (previously defined under the *queues* root element) can be referenced. Conversely, for atomic deploy mode and/or APIs package mode another step of the pipeline can be referenced. Each input and output queue defined might have a condition associated to identify when to redirect the data to this element. If no condition is associated to the queue, it always will receive the data. For instance, if multiple outputs, without conditions, are defined all of them will receive the data flow.

### Types of pipelines

**Fig. 14:**
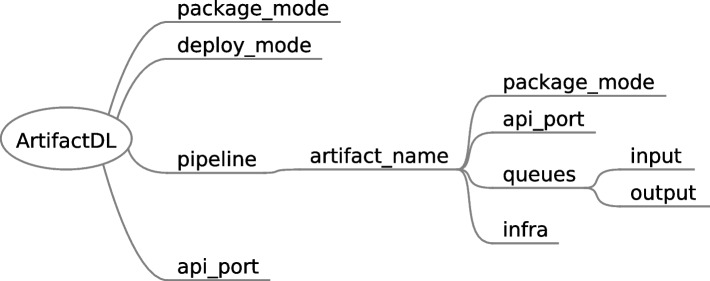
ArtifactDL elements to support different types of pipelines

ArtifactDL integrates elements to dictate the type of pipeline to be built (see Fig. 14). Under *ArtifactDL* entity *package_mode* indicates if the steps of the pipeline must be deployed wrapped in an API or in streaming. For the API mode, specific port must be set to expose the API by using the *api_port* element under the root element for atomic deployments or selecting the specific port for each artifact at the step level for distributed deployments. For streaming, specific input and output queues must be defined to adequately build the necessary connectors. Moreover, a more fine-grained control can be achieved indicating the *package_mode* at the step level which will have prevalence over the root level.

*deploy_mode* element allows to indicate whether the pipeline is deployed in *atomic* or *distributed* mode. Atomic mode should deploy the whole pipeline embedded in a container and distributed mode is devoted to deploy each step of the pipeline in separate containers. Then, whether the containers in distributed mode are deployed in the same machine or not is driven by the infrastructure set in the *infra* element of each step.

Additionally, regardless the specific package mode configuration of each element of the pipeline, at the pipeline step level, the output queue can be configured to use a data space producer connector. Thus, the pipeline can be exposed in order to participate as a a service in a data space ecosystem. For this purpose, the connector to be used in the output must be set as well as a *self-descriptor_url* property targeting a file with the specific configuration of the service to offer through the connector.This way, data sovereignty and interoperability are promoted.

### Artifacts support

In PADL, an analytic pipeline could be composed of different chained artificial intelligence models. However, in real world scenarios analytic pipelines are generally composed of code blocks which can be utilised for performing pre-processing or post-processing for dealing with tasks like standardization, feature engineering, data cleaning, etc. Therefore, besides artificial intelligence models, ArtifactDL supports the inclusion of steps of code in a pipeline. This enables the possibility to create (see Table 1):

1. More traditional DevOps pipelines to deploy a code artifact in a production environment after being validated in a testing environment.
2. DataOps pipelines to automate the different transformations and operations to be done over data. These transformations are also managed by a code artifact.
3. Training pipelines since the preparation, feature engineering, training and evaluating steps can be decomposed into a sequence of steps programmed in a specific code or a set of code projects.
4. Inference pipelines. The ability to set blocks of code enables the creation of full analytic pipelines with all the steps required.
5. IaC pipelines. For instance, the creation and configuration of a virtual machine could be accomplished by executing a block of code that triggers a Vagrant file to create the virtual machine and then, another one that executes an Ansible role to configure it with the necessary libraries and services.

**Fig. 15:**
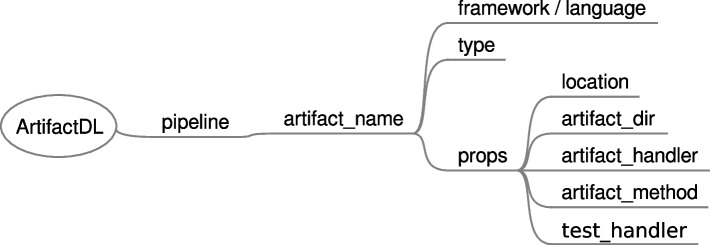
ArtifactDL syntax for managing different types of artifacts

As a generalization, we can define that a pipeline is composed of a set of artifacts that can be of the type artificial intelligence model or code type. Figure 15 shows the properties included for this purpose. *Framework* property is only used for models with the objective of identifying the specific AI framework utilised to create the model like TensorFlow [49], Scikit-learn [48] or Pytorch [91]. Conversely, *language* defines the programming language in which a software has been coded, for instance Python, C++ or Java. Then, *props* is designed to unequivocally identify the artifact location and the function to execute which includes:

- *Location*: it corresponds to where the artifact is stored. For instance, the path to a local or remote folder or a GIT [92] repository.
- *artifact_dir*: the model to execute or the entry point of a software are not always allocated in the project root folder. Consequently, this property targets such required location.
- *artifact_handler*: in the case of models, it corresponds to the serialised model file inside the project. Conversely, for software projects, it is required a function that is able to receive an input, orchestrate the necessary tasks to execute and return an output. Thus, a dedicated software solution could adequately wrap, for example, such function in a REST API or with input and output queues for data streaming. This property corresponds to the file where such function is allocated.
- *artifact_method*: the name of the function to execute.
- *test_handler*: to identify the testing class.

### Training pipelines

**Fig. 16:**
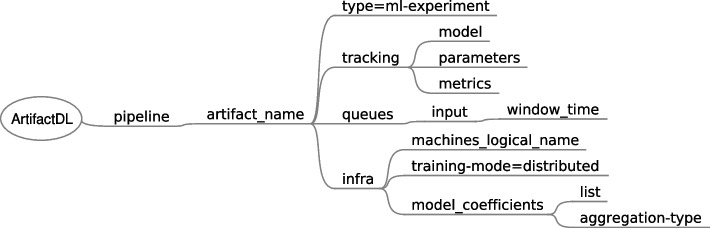
ArtifactDL syntax specific for training artifacts

As mentioned in the previous section, the train stage of a ML model, in essence, is conducted by a software program which uses specific ML libraries or frameworks to accomplish this task. As a consequence, in ArtifactDL a training step for an analytic pipeline could be directly treated as a code artifact. However, it incorporates additional features to boost training steps and a specific artifact to wrap them has been conceptualised, called *ml-experiment*. The summary of these features are explained below and shown in Fig. 16 which presents the elements introduced in the ArtifactDL schema to support these features:

- Experiments tracking. The output of an ML process can be composed by the resulting model trained alongside the results of the validation stage and the parameters used for the training. These elements can be captured and stored in a model registry augmenting the model with this relevant meta-information. In this regard *tracking element* has been defined to identify the elements to track: models, parameters and metrics. In this way, code to track these elements could be automatically integrated with the original code. For instance, this functionality can be accomplished by making use of the MLFlow library [93].
- The possibility of tracking models enables to deploy those resulting models in a production environment. For this purpose, a secondary ArtifactDL pipeline definition can be prepared describing an inference pipeline which can be deployed when a specific condition is satisfied. For instance, when a performance metric is higher than a specified threshold. In order to fulfill this objective, monitoring rules can be set (see “Monitoring support” section).
- Continuous training: the data used to train the model can be directly identified in the program code to execute. For example, targeting a specific CSV file near the code. However, *queues* mechanism can also be utilised to define the input of the training step, for instance, identifying a specific database and the required access mode or being connected to a streaming source enabling a continuous training process governed by specific parameters. Element *time_window* under *input* and *queues* can be used to periodically get the new data in the queue. Initially, all the available data should be retrieved and then, periodically based on the time window specified to promote continuous training.
- Distributed training: the training step can be executed in parallel in a set of machines dividing the input dataset among them. For this aim, concrete aggregations between the involved machines must be applied after each training iteration to synchronized specific model intermediate results (such as weights). *infra* element has been augmented to admit a list of machines instead of a single one. In addition, *training-mode=distributed* indicates the machines that should be used to perform a distributed training. Then, *model_coefficients* includes a list for identifying the specific relevant coefficients and *aggregation-type* defines the operation to perform when synchronizing the coefficients. It should be noted that the AI algorithm used must be compatible with distribution.

### Monitoring support

Artifact monitoring is key to verify the adequate behaviour after the deployment stage. For example, model monitoring has become a key stage of the MLOps life-cycle to quickly identify model drifts [94] causing a deterioration in the model performance. Besides model monitoring, the incoming data should also be monitored because data not coming in the ranges of the used for the training model could also cause a concept drift [95]. When detecting this worsening in the model performance, a specific action should be triggered such as a notification alarm or a retraining and redeployment of the model. In addition, the underlying infrastructure should be monitored to guarantee that the artifact is being executed under ideal conditions. If problems arise in the infrastructure, again a specific action should be conducted such as an alarm or deploying the artifact in other machine. For example, this functionality is achieved by container orchestrating technologies like Docker Swarm [59] or Kubernetes [58] that redeploys a container in a distinct machine of the cluster when a machine is not in the desirable state.

**Fig. 17:**
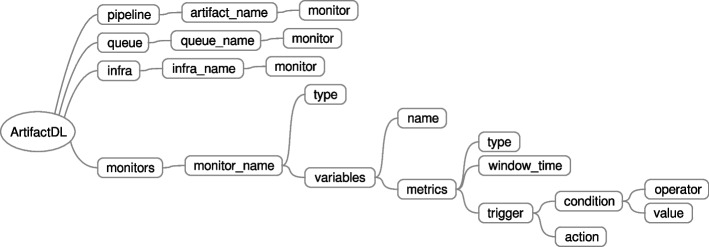
ArtifactDL syntax specific for monitoring

In ArtifactDL three monitoring types are supported: model, data and infra. Each monitoring rule is defined under the tag *monitors* which enables defining the type, the variables to watch, the metrics to consider and the actions to perform by using the tag *trigger*. Then, the defined monitored rules can be attached to artifacts, queues and infra elements by using a dedicated *monitor* tag under each of these elements (see Fig. 17). Consequently, a pipeline step with a model, the data flowing in a queue and the utilised machines can be monitored.

### Governance

The previous monitoring “Monitoring support” section can be considered as part of a governance mechanism. As highlighted in [42], data monitoring to detect a data drift is directly related with data quality concepts such as bias detection and, in turn, model drift detection is related with model quality when detecting changing patterns in the model output (or indeed in the output of a code block). In addition, monitoring mechanism can be used to carry out other data quality aspects such as detecting biases, missing data, duplicates or average, standard deviation, maximum and minimum values not matching a threshold or distribution. In consequence, specific triggers can be configured to set default values for missing data, remove duplicates or discard and store anomaly values over a period of time.

**Fig. 18:**
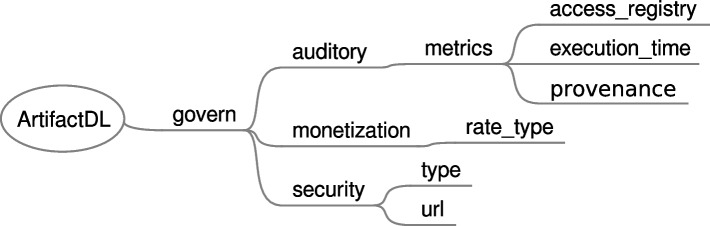
ArtifactDL syntax for addressing governance

Besides quality aspects, when defining analytic pipelines other governance mechanisms should be taken into consideration which are shown in Fig. 18. For instance, *auditory* tag enables the activation of a set of metrics:

- *access_registry* to define that the number of executions requested to an artifact must be tracked alongside a timestamp. If a security mechanism is enabled, the involved user is also registered. This serves to identify the use an artifact is subjected to and, in addition, it is necessary when monetization per use rate is enabled. Technically, a piece of code should intercept the requests made to the audited artifact to store the corresponding information.
- *execution_time* indicates that before and after the execution of an artifact the corresponding timestamps must be registered to calculate the execution time. This property can serve to analyse the performance of the artifacts over a period of time and also can be useful for monetization services.
- When *provenance* is activated, additional meta-information must navigate with the data flowing through the artifacts to keep track of the previous services that have interacted with the original data registry.

Monetization can be enabled to specify that the use of an artifact requires a payment. Diverse rate types can be specified like a flat rate or a pay per user approach. As mentioned, the latter necessitates to enable some auditory mechanisms to keep track of the use of the artifact as well as a security service.

Finally, without security enabled any anonymous user can make use of an artifact. Conversely, with security only authenticate users are allowed to use them. Then, depending on the type of the security service authorisation rules and more specific complex policies can be applied. *url* property is set to link a security management systems like Keycloak [43]. Therefore, if for instance, this will be the case, the system dealing with this syntax must automatically create APIs or connectors with Keycloak required specifications included and, in turn, users should provide an access token or similar when using the artifacts.

## Assessment across application scenarios and by expert-judgment

In the context of the growing complexity of intelligent systems and the increasing need for specialized tools to address existing challenges and automate methodologies like MLOps and DataOps, Domain-Specific Languages such as ArtifactDL have emerged as a promising solution. This section presents an evaluation of this language within the framework of its use in the European Pliades project. It aims to rigorously and comprehensively assess the usefulness and efficiency of the DSL developed specifically for modeling data product pipelines and the training and inference of models fed with such data. To address these challenges and provide a thorough assessment, we have designed a dual approach that combines practical implementation based on scenarios with expert evaluation. These two approaches will allow us to obtain a holistic view of the capabilities and potential impact of DSLs. On one hand, implementation in real-world scenarios, presented in “Use case scenarios” section, provides tangible evidence of their applicability and versatility. On the other hand, expert evaluation, explained in “Experts evaluation” section, offers a critical and substantiated perspective on their benefits and potential limitations compared to alternative methods or their non-use.

### Use case scenarios

This section presents an scenario to better show the ArtifactOps methodology in action and a set of modifications over it to expose the flexibility of ArtifactDL. The goal is to replicate the implementation in diverse usage scenarios, and to this end, Artifact DL will be implemented in a variety of carefully selected usage scenarios. These scenarios range from a real-world use of model inference in the agricultural domain to the combination of two European projects, Illumineation and Pliades, where the former provides the data and the latter uses and shares it to generate models in a common “Data Space” ecosystem. This allows us to evaluate the language’s adaptability and expressiveness in the face of problems of diverse nature and complexity. This approach will allow us to empirically demonstrate the flexibility and power of ArtifactDL in diverse, real-world contexts.

#### Scenario 1- real-life use case for model inference in the farm domain

Islam et al. [96] presents a real scenario which uses an online web application aimed at assisting farmers in identifying diseases of plants by analyzing photos of the plant leaves. To this end, this system integrates a machine learning model devoted to predict crop disease based on deep learning techniques. This section presents how to solve this particular scenario with ArtifactOps methodology and ArtifactDL.

The application of the methodology starts with the *definition phase*. During the problem statement the objective and required functionalities have already been established. Regarding such ecosystem of diverse software clients utilised by the farmers, it is decided to offer the service through a REST API which is suitable for web applications.

Then, the application promoters could decide to design the following pipeline:

1. Capture the input: the REST service must be able to receive the new photos submitted by the farmers that will be analyzed to detect crop diseases. Consequently, the user input must be adequately modeled to ensure the interoperability.
2. Discard data with failures finishing the flow or let adequate data pass to the following step (a code artifact).
3. Normalize the data to be prepared for the input model (a code artifact).
4. Execute the prediction model (a model artifact).
5. Provide a response with the prediction output to the client.

Regarding inputs and outputs, each component must be compatible with the following one and the first input is the one from the user and the last output is the response with the prediction which should process the web application. Furthermore, Python is used to code the artifacts, the AI framework to train the prediction model for detecting crop diseases is not specific in [96], but let’s suppose that is TensorFlow [49] and that the deployment mode will be atomic since there is no benefit in offering any step as a isolated service and it avoids expending money in additional machines. Consequently, it is decided to create a virtual machine in a cloud provider to deploy the service. As tests is concerned, unit tests for each code artifact are designed, the model performance is defined to be higher than the 86% and a set of tests to verify the whole pipeline are defined.

Then, specific users and associated permissions are defined to enable the security and the company decides not to invest more money in Governance in this stage since, among other things, the service is not going to be monetized in the short term.

The team decides to monitor a set of aspects 1) the model performance to identify if there is a degradation in the prediction of crop diseases, 2) the input data to check whether is different from the distribution used in the training process and, consequently, the model should be retrained to deal with the new input patterns and 3) the virtual machine to minimize the service down-times.

Finally, the package mode has already been established as *API*, the deploy mode to *atomic* and the flow has also been defined.

Then, in *artifacts creation phase*, the promoters prepare the diverse code artifacts with the correspondent unit tests and train the model with different parameters until getting a performance higher that the 86%. Finally, all the artifacts are uploaded to a GIT repository.

During *infrastructure management phase* they must create the virtual machine in the cloud. For this purpose, an IaC pipeline is decided to be created. Consequently, they follow this ArtifactOps methodology. Summarizing, the pipeline is composed of two code artifacts which after passing successfully the corresponding tests are executed to provide the infrastructure. The first one a Vagrant file to programmatically create the virtual machine and the second an Ansible playbook to properly configure it.

The corresponding ArtifactDL pipeline is created in the *pipeline creation phase* (See Listing 1 where some elements have been obviated to minimise the space and for being redundant). Package and deploy modes are set to *API* and to *atomic* respectively to create an API with the whole pipeline. In addition, the resulting API will be embedded into a Docker container to ensure the portability between diverse operative systems. The port where the API is going to be exposed is also set. The pipeline is composed of the three artifacts. 1) The *discard_incorrect_data* code block established two outputs driven by a set of conditions. The first output, without an output name, for finishing the flow and the other to continue it. 2) The *normalization* code step and 3) the *predict* model with its corresponding characterization. It can be observed that only the queues of the artifacts are defined because in the REST API mode the input and output are directly considered the pipeline artifacts. Regarding infrastructure, two machines are defined for testing and production environments which are accordingly labelled to properly manage the deploys. *monitors* element provides also an example of the rule defined to watch the performance of the model. Finally, *govern* element establish a rule to utilise Keycloak as security service where the users and their permissions must be defined.

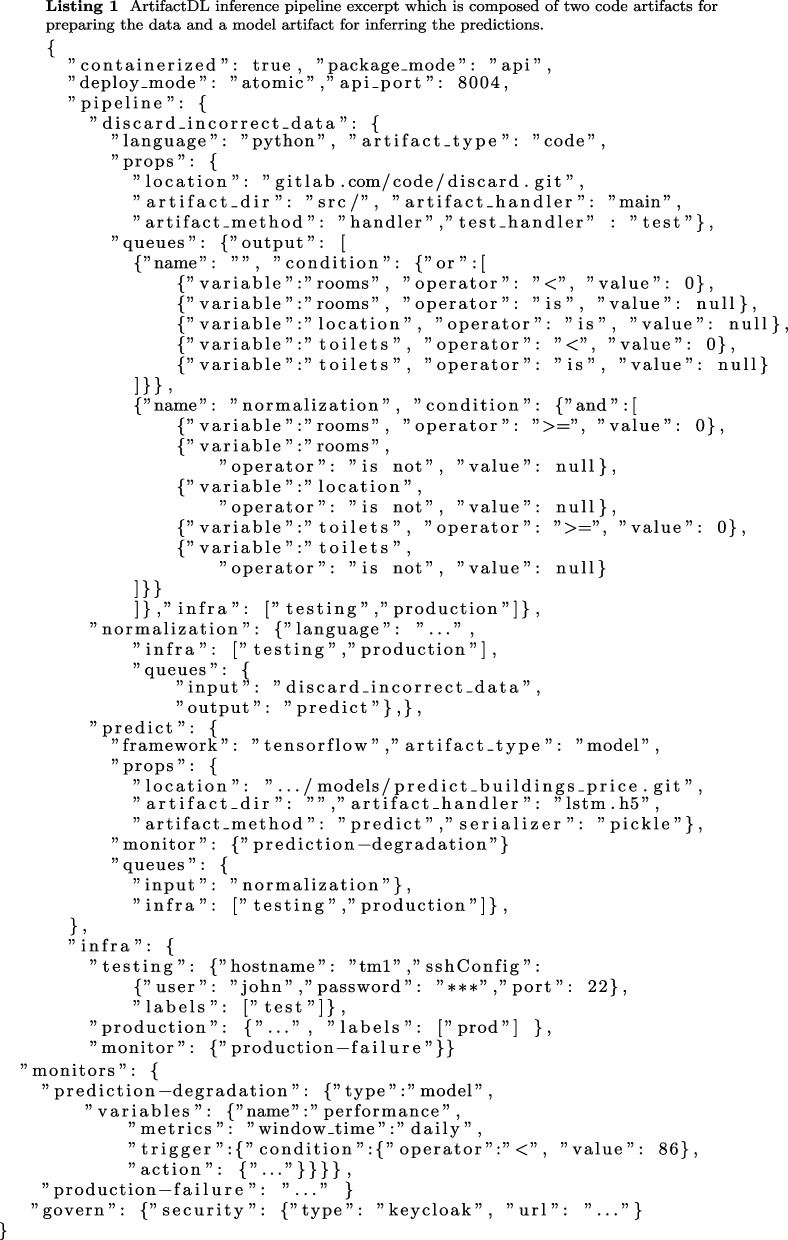



In *test and fix phase*, the application is deployed in the testing environment and the artifact unit tests are executed. If there would occur some errors, the developers would fix them. Next, the whole pipeline is executed to test its flow. Finally, when all the tests are successful the testing and fix phase finishes.

Then, the pipeline is deployed in the production environment during the *deploy phase* and the farmers could make use of the service by submitting their own plant photos in order to predict crop diseases. It is worth noting that for the deployment in both testing and production environments an ArtifactDL-compliant tool must be utilised. Works like [6] or [30] covers it in some extent but new developments should be done to deal with all the functionalities.

For the *monitor and govern phase* the pipeline is already deployed, the only task to be done is to be conscious of the monitoring and governance rules which can cause specific actions.

#### Scenario 2- collaboration between Pliades and Illumineation projects: training, inference and data space scenario

On the one hand, in the context of the European illuMINEation project a multi-layer (edge-fog-cloud) IIoT platform was developed for the mine domain which enables the processing of data an ML algorithms in a distributed fashion [97]. On the other hand, Pliades European project [18] is focused on AI life-cycles and its integration with data spaces. This scenario is aligned with both projects. In the IlluMINEation project, it was captured data from intelligent rock-bolts sensors which measure the rock mass deformation.

In the *design phase*, we decide to utilize this information to train periodically a model to detect anomalies. After training, this model, aligned with Pliades project, we want to offer it as a service through a data space ecosystem once its performance was good enough. The result of this process is a specific version of a model artifact provided in a data space ecosystem which has been trained by using data received from intelligent rock-bolts. To this end, four distributed artifacts in an edge-fog-cloud layer are defined and created in the *artifacts creation phase* 1) the one to discard the bad readings (edge layer), 2) one for data normalization (fog layer), 3) the code for training the model (cloud layer) and 4) the model deployment for inferring new anomalies (cloud layer).

For the *infrastructure management*, illuMINEation infrastructure is utilized. As mentioned, in [97] a gateway with Python support is provided for the edge layer. The fog is composed of a cluster of machines with big data capabilities for each mine. Finally, there is a cloud with more powerful hardware resources to aggregate data from all the mines and to deal with resource-demanding processes. Moreover, a specific machine for deploying the tests will be utilized.

After creating the artifacts and the infrastructure, the ArtifactDL document is created in the *pipeline definition phase.* Listing 2 provides an excerpt of this pipeline for this scenario. Being a distributed streaming pipeline, input and output MQTT queues have been defined for the communication between the first two steps and steps two and three. The output of the third artifact is a model that is uploaded to a model registry once its performance satisfy a established criterion by using a MLFlow tracking server. Finally, step 4 takes such model and exposes through a data space connector as a data space producer service. The monitoring of the training stage allows to deploy a pipeline when certain condition is satisfied. This pipeline re-deployment enables the automatic update of the whole inference pipeline of the previous scenario. Next, the pipeline passes the *test and fix*, *deploy* and *monitor and governance phases* to complete the process which will be iteratively executed.

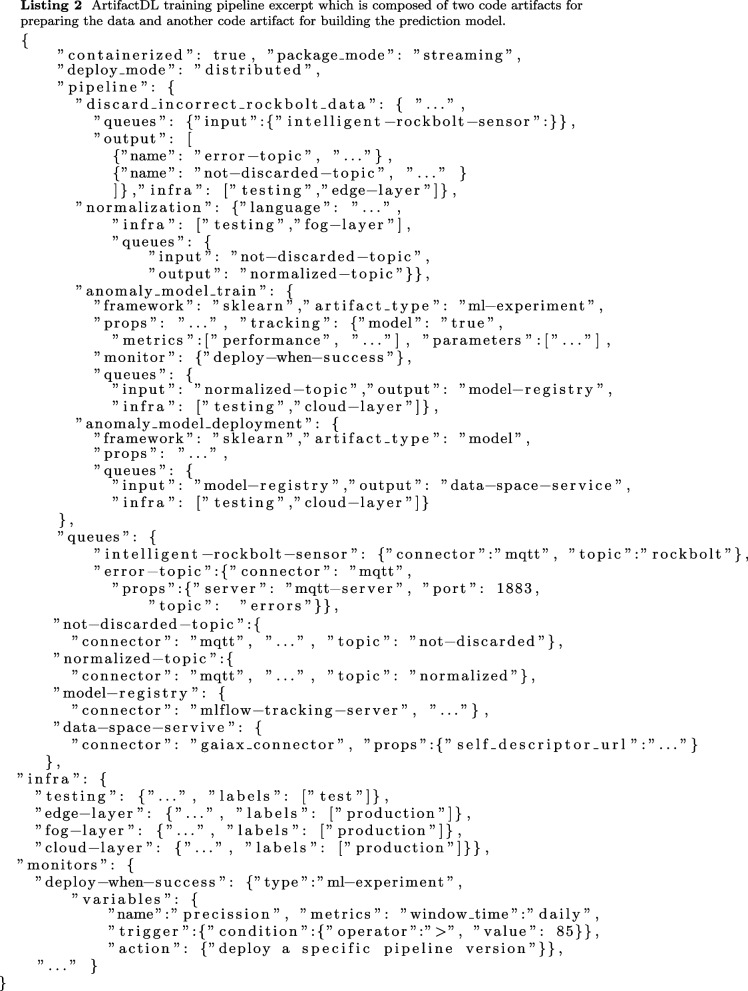



### Experts evaluation

This section presents an experts evaluation conducted to validate the usefulness of ArtifactOps methodology. In parallel with the scenario-based evaluation already presented, a committee of experts, composed of researchers participating in the Pliades project, will conduct a comprehensive evaluation of the proposed methodology. This evaluation will be conducted through a series of tests designed to measure the effectiveness, efficiency, and added value of the Artifact DL tool compared to traditional approaches or the absence of a specialized tool. The tests will address aspects such as the learning curve, developer productivity, the quality and expressiveness of the resulting specification, and its legibility and ease of extension and maintenance, among others. Therefore, next subsections offers its preparation, results and a discussion.

#### Testbed preparation

First, the following question has been designed to confirm the adequacy of the evaluator proposed. Consequently, evaluators with an score less than three in this question are discarded.

1. What is your previous experience with methodologies like DevOps, DataOps, or MLOps? (1: None, 5: Expert)

Subsequently, below five questions have also been defined to better analyze the usefulness of ArtifactOps and ArtifactDL:

2. Do you think a unified methodology like ArtifactOps which enables the concept of pipeline and the merge of different artifacts (code, data, models, infra management) could address the current challenges in your work? (1: Not at all, 5: Definitely yes)
3. How well do you think ArtifactOps and ArtifactDL can ensure interoperability between different types of pipelines (DevOps, DataOps, MLOps)? (1: Not at all, 5: Extremely well)
4. Due to the possibility of composing pipelines of different artifacts developed by different roles, how do you think ArtifactOps and ArtifactDL could facilitate the integration and collaboration between different teams in your organization? (1: Will not facilitate, 5: Will greatly facilitate)
5. How do you think ArtifactOps could help your organization scale and adapt to changes in the environment? (1: Will not help, 5: Will greatly help)
6. How difficult is it to implement and adopt ArtifactOps in your organization? (1: Very difficult to implement, 5: Very easy to implement)

Finally, a summarized description of ArtifactOps and ArtifactDL were provided in a document alongside the questions. Then, such document and the current version of this research article (in case some expert may like to get a better comprehension of the approach) were submitted to a group of fourteen experts in the area. Concretely, people with background in at least three of the unified methodologies: DevOps, DataOps, MLOps and IaC. A week was set as the deadline to answer the pool of questions.

#### Experts results and discussion

**Fig. 19:**
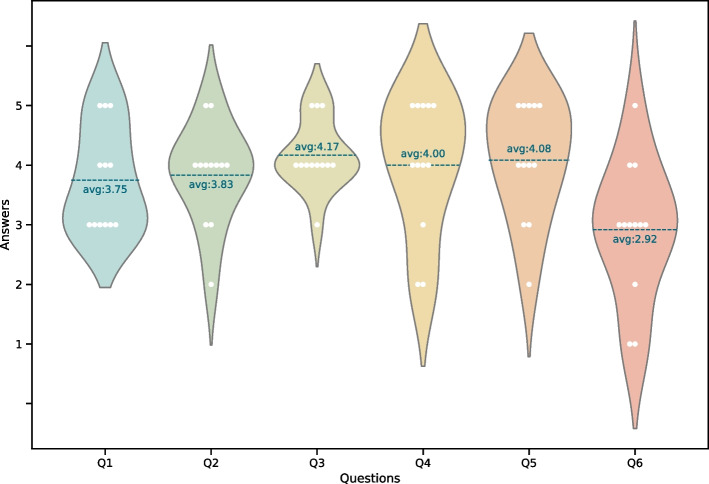
Violin graph where it can be seen the data distribution and in which category are located each registry

**Fig. 20:**
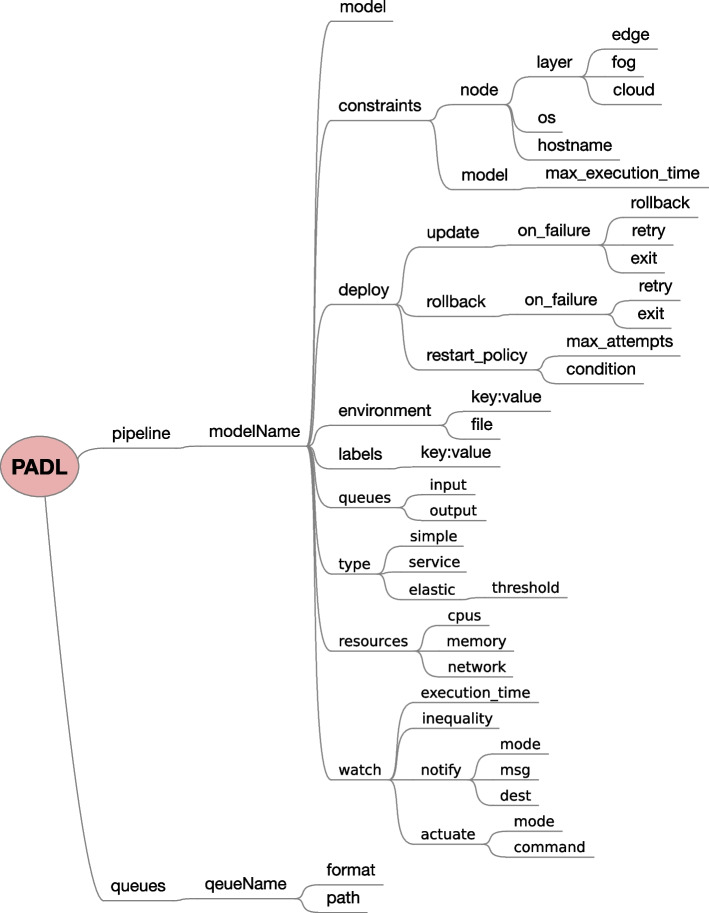
Elements included in PADL syntax for defining analytic pipelines

After such week, one expert did not provide answer and another one marked with 2 the filter question. As a consequence, twelve answers were considered. Figure 19 presents violin graph where all the answers can be appreciated as well as the average and the data distribution.

First, it is considered a significant good expertise level in the evaluators (Q1). As mentioned, only one of the responders was discarded and the average expertise of the rest of the evaluators is 3,75.

Then, in the evaluation questions (Q2-Q6) all of them are with a score higher the 2,9 of 5 which is a good indicator to keep promoting the approach.

Question Q2-Q5 obtained significantly good results, all above 3,8. Q2 with 3,83, Q3 with 4,17, Q5 with 4,00 and, finally, Q5 with an score of 4,08. This clearly validates the hypothesis that ArtifactOps methodology alongside ArtifactDL can promote the heterogenrous pipeline development (Q2), the interoperability (Q3), the integration among teams (Q4) and that can help in scaling and adpating changes in the environment (Q5).

Although the overall evaluation results are largely positive and support the central hypothesis of this work, the lower score obtained for Q6-related to the feasibility of adopting the methodology within organizations (mean score: 2.91)-indicates a key concern around perceived adoption complexity. This suggests that future efforts should focus on reducing entry barriers and supporting organizations in this transition. As preliminary strategies, we identify three critical actions: (1) fostering cultural acceptance through targeted training programs that clearly communicate the methodology’s benefits and practical implications; (2) identifying and implementing mechanisms to reduce the learning curve, such as simplified on-boarding materials and usage guidelines; and (3) developing a mature tool-chain that streamlines the design, validation, and deployment of ArtifactOps pipelines. These steps are essential to facilitate real-world adoption and maximize the methodology’s impact.

### Threats to validity

The evaluation is susceptible to internal validity threats, such as selection bias (expert panel $n=12$ may not represent the broader DevOps/DataOps/MLOps community) and a theoretical focus limiting practical effectiveness measurement. External validity threats include limited generalizability to organizations without prior experience in unified methodologies or smaller teams with constrained resources, as well as reliance on the current ArtifactDL tool version and lack of direct comparisons to alternatives (e.g., hybrid workflows). Mitigations for internal validity involve diversifying the evaluator pool, incorporating qualitative feedback, and validating findings through real-world case studies. For external validity, future work should test the methodology in diverse organizational contexts and benchmark it against existing tools.

Despite these threats, the evaluation provides a robust foundation for ArtifactOps and ArtifactDL. The high expertise level of evaluators (average Q1 score: 3.75) and strong results for core questions (Q2-Q5, all >3.8) support the methodology’s relevance. While the lower Q6 score (adoption complexity) reflects a practical challenge, the current validation-based on expert consensus and targeted design-is sufficient to justify further research and application, especially as proposed mitigations (training, tooling, and cultural adaptation) are already outlined.

## Conclusions

This papers presents three different contributions. First, key aspects when defining diverse types of pipelines (CI/CD, IaC, data, train and inference/analytic) are deeply examined and contrasted with the existing works in background section (“Background” section): the selection of the artifact type, the definition of the purpose of the pipeline which derives in a specific type of pipeline, the characterization of the input and output, the definition of package and deployment modes and the consideration of governance rules.

The second contribution is the ArtifactOps methodology which aims at unifying the DevOps, MLOps and DataOps phases, while considering IaC pipelines, in a unique methodology. In addition, it considers not only artifacts but also the creation of pipelines capable of merging different artifacts. For this purpose, at the beginning, “ArtifactOps methodology” section shows a comparison of the distinct phases of these paradigms to clearly show their differences and likeness and afterwards, the methodology is presented demonstrating that they have more aspects in common than differences. Therefore, a single methodology, divided into seven phases, addresses these paradigms, in an unified fashion, by generalizing some concepts or making them wider. This section also emphasizes the cultural changes recommended to enhance the adoption of the methodology within companies. Finally, “ArtifactOps methodology” section includes two subsections: one to identify the necessary tool ecosystem to better promote the methodology, and the other to outline its versatility in complex scenarios such as critical systems, as well as in less restricted environments.

The last contribution consists in the ArtifactDL pipeline definition language, which supports all the types of pipelines defined in Table 1 (CI/CD, IaC, Data, train and inference). Among its features, it can be highlighted: 1) detailed infrastructure support, 2) flexible queues, 3) support for diverse types of pipelines and package and deployment modes, 4) support of not only ML models but code artifacts and an specialization of them (ml experiments), 5) monitoring rules, 6) governance consideration and 7) the possibility of providing the result of the pipeline in a data space ecosystem by offering it by means of a data producer. “Background” section has stated the main research works which served as the foundation of ArtifactDL and ArtifactOps as a background. Moreover, in “Related work” section the related works have been examined reaching to the conclusion that there exists a gap for this work. In addition, two different real scenarios have been detailed in “Use case scenarios” section to better explain the utilization of ArtifactOps methodology and ArtifactDL pipeline definition language and to demonstrate its versatility when defining different types of pipelines. Finally, “Experts evaluation” section have discussed an expert evaluation conducted to validate the suitability of the research where all the scores obtained are high enough to impulse the approach.

As a summary, we strongly believe that the present proposal can help software developers, DevOps and data engineers and data scientists to approach the problem of merging various diverse artifacts from a different perspective, instead of having to use a set of different paradigms. At least, this research paves the way to unify diverse widely used XXOps paradigms and, consequently, simplifying its application and reducing its complexity. The tool ecosystem defined in “Tooling ecosystem for ArtifactOps” section to boost the adoption of both ArtifactOps methodology and ArtifactDL will be carried in the future work.

## Data Availability

No datasets were generated or analysed during the current study.
